# HIV-1 cellular and tissue replication patterns in infected humanized mice

**DOI:** 10.1038/srep23513

**Published:** 2016-03-21

**Authors:** Mariluz Araínga, Hang Su, Larisa Y. Poluektova, Santhi Gorantla, Howard E. Gendelman

**Affiliations:** 1 Department of Pharmacology and Experimental Neuroscience, College of Medicine; University of Nebraska Medical Center USA

## Abstract

Humanized mice have emerged as a testing platform for HIV-1 pathobiology by reflecting natural human disease processes. Their use to study HIV-1 biology, virology, immunology, pathogenesis and therapeutic development has served as a robust alternative to more-well developed animal models for HIV/AIDS. A critical component in reflecting such human pathobiology rests in defining the tissue and cellular sites for HIV-1 infection. To this end, we examined the tissue sites for viral infection in bone marrow, blood, spleens, liver, gut, brain, kidney and lungs of human CD34+ hematopoietic stem cell engrafted virus-infected NOD.Cg-*Prkdc*^*scid*^ *Il2rg*^*tm1Wjl*^/SzJ mice. Cells were analyzed by flow cytometry and sorted from species mixtures defined as CD34+ lineage negative progenitor cells, CD14+CD16+ monocyte-macrophages and central, stem cell and effector memory T cells. The cell distribution and viral life cycle were found dependent on the tissue compartment and time of infection. Cell subsets contained HIV-1 total and integrated DNA as well as multi-spliced and unspliced RNA in divergent proportions. The data support the idea that humanized mice can provide a means to examine the multifaceted sites of HIV-1 replication including, but not limited to progenitor cells and monocyte-macrophages previously possible only in macaques and human.

A principal hurdle in the treatment and perhaps eradication of HIV infection rests in finding sites of both persistent and latent infection that occurs despite antiretroviral therapy (ART)^1^,^2^. Such cells are not eliminated by immune surveillance and readily can go undetected. Infection commonly re-emerges during activation, cell-to-cell contacts or during terminal cell differentiation^3^,^4^. Continuous infection occurs in long-lived CD4+ T cell subsets along with a range of myeloid lineage cells that serve as secondary viral targets^5^,^6^,^7^,^8^,^9^. With changes in time and population dynamics, defining each infected cell type is imperative in any viral treatment of inevitable elimination strategy^10^,^11^,^12^. Notably, viral persistence in diverse anatomical sanctuaries, such as lymph nodes, and the gastrointestinal, genitourinary and the central nervous systems (CNS), are notable obstacles to effective therapeutic, immunization and eradication strategies^13^. This underscores operative physiologic and anatomic barriers and a common lack of pharmacologic and immunologic penetrance^14^. With these biologic facts in hand, defining sites for continuous and active virus persistence, despite avid host innate and adaptive antiviral immune responses, remains critically important. Indeed, high viral loads are readily seen even following massive depletion of CD4+ T cells, the principal virus-targets. Persistent low-level infection in monocyte-macrophages may contribute to the viral load^20^. Thus, defining the biological relevance of each HIV target in its natural anatomic sites in relevant animal models is imperative. This includes, but is not limited to, studies of the proportions of central and transitional memory CD4+ T and myeloid cells and viral decay rates, if a functional viral cure can be achieved^16^,^17^,^18^,^19^.

During the past decade the use of humanized mice to study HIV-1 biology, virology, immunology, pathogenesis and development of therapeutics has served as a potent alternative to more-well developed animal models for HIV/AIDS including simian immunodeficiency virus (SIV) infected rhesus macaques^15^,^20^,^21^,^22^,^23^,^24^,^25^. The model’s ability to form a functional immune system and sustain longer-lived cells makes it a suitable model to study the dynamics of virus-host cell interactions^26^,^27^,^28^. Lymphocyte depletion and viral end-organ disease are additional features of this animal model^29^,^30^. Since CD34+ hematopoietic stem cell (HSC)-transplanted NOD.Cg-*Prkdc*^*scid*^ *Il2rg*^*tm1Wjl*^/SzJ (NSG) mouse is the simplest and most accessible model for the studies of viral infection compared to the complexities of transplanting human liver and thymic tissues with autologous CD34+ HSC needed to generate BLT mice, we took advantage of CD34-NSG model for the investigation of viral distribution in tissues and sorted cell populations to define virus-cell interactions. Using humanized mice, virus infected human cells from bone marrow, blood, spleen, gut, lung, kidney and liver were quantified by measuring viral DNA and RNA. This assessed the levels of infection in CD4+ T cell subsets and monocyte-macrophages during persistent infection. The sites of viral infection in human immune system engrafted mice were found to be dynamic with changes seen over time. This included the actual cell subsets that serve as viral targets. Interestingly, both monocyte-macrophage and lymphocyte lineage cells were sites of persistent viral infection. Viral levels were found dependent on tissue sites. Central and effector memory T cells together with progenitor and myeloid cells are cellular sites of virus replication. Interestingly, the cell type distribution changed with time and levels viral infection. CD34 mice are completely relevant for investigation of infected T cell, macrophage and progenitor stem cells operative during HIV infection and reflect the dynamics of viral infection that occurs in a human host. Thus, the model is relevant for the studies of disease biology and the means to affect reduction of residual virus. Our findings revealed specific cell subsets for further therapeutic and viral elimination studies.

## Results

### Defined infection sites of HIV-1 in tissues and cells

**Figure 1:**
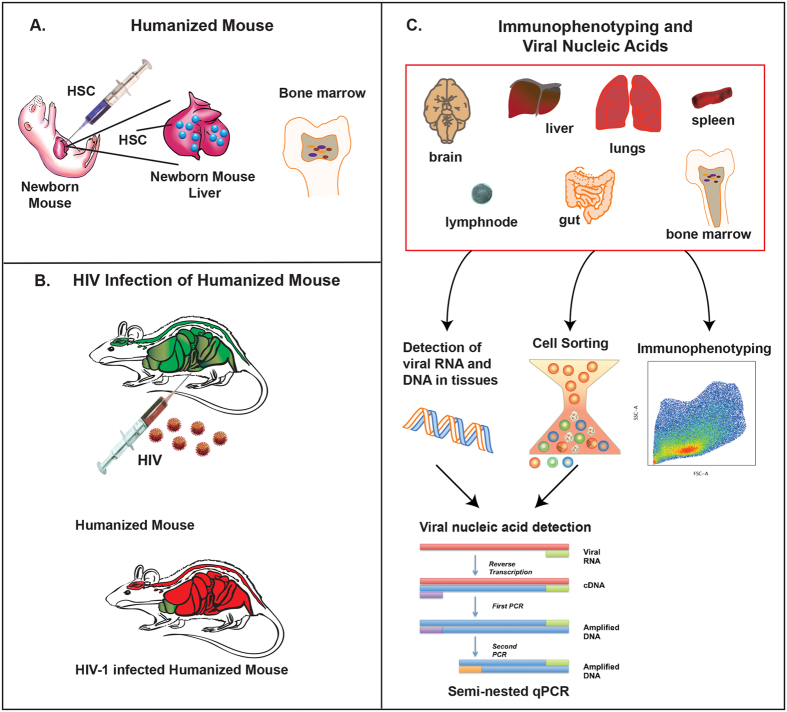
Experimental approaches for tissue and cellular target testing of HIV-1 infected humanized mice. (**A**) New-born NSG mice were transplanted with human HSC from cord blood. (**B**) After three months of human cells transplantation, mice were infected with HIV-1. (**C**) At 5 weeks post HIV-1 infection, several tissues such as bone marrow, spleen, lung, gut, brain, kidney and liver were collected for RNA and DNA isolation for the detection of HIV-1 using a semi-nested qPCR method. In addition, at 11 weeks post infection, spleen and bone marrow cells were isolated and sorted for collection of Lin-CD34+, CD14+ CD16+, T_SCM_, T_CM_, T_EM_ and T_REG_ populations. Sorted cells were used for the extraction of RNA and DNA, and viral quantification in each cellular subsets was determined by semi-nested qPCR. Moreover, cell phenotyping was determine by flow cytometry for the duration of the study where blood, spleen and bone marrow were collected for the identification of, memory CD4+ T cells, regulatory CD4+ T cells, Lin- CD34+, monocytes/macrophages CD14+ CD16+ and dendritic cells CD14− CD16+, as explained in methods.

Our laboratory has previously used humanized mice for studies of HIV-1 pathogenesis and antiretroviral drug delivery^15^,^21^,^22^. In the current report, immune deficient mice reconstituted with human CD34+ stem cells (Fig. 1A) were used to survey the distribution patterns of virus infected cells. Hematopoietic stem cells were isolated then injected into the liver of irradiated new born mice. Human cell engraftment was assayed at twelve to fifteen weeks by flow cytometry of peripheral blood. Reconstituted animals were infected with 10^4^ tissue culture infectious dose_50_ (TCID_50_) of HIV-1_ADA_ injected intraperitoneally (Fig. 1B). Virus was assayed in plasma starting from 4 weeks post-infection (Supplementary Table 2); and viral nucleic acids were assayed in recovered bone marrow, spleen, lung, gut, brain, kidney and liver by semi-nested quantitative polymerase chain reaction (qPCR) (Fig. 1C). Lineage-1 negative (Lin-) CD34+ cells, monocyte-macrophages and memory and regulatory CD4+ T cells were immune sorted from bone marrow and spleen, then assayed for viral nucleic acids. In addition, for the identification of specific immunocytes, flow cytometry was performed from five to fourteen weeks post infection. Uninfected mice reconstituted with the same donor HSC were included for comparison at each time point. A group of at least five animals was euthanized for each time point (Fig. 1C).

### HIV-1 tissue target sites

**Figure 2:**
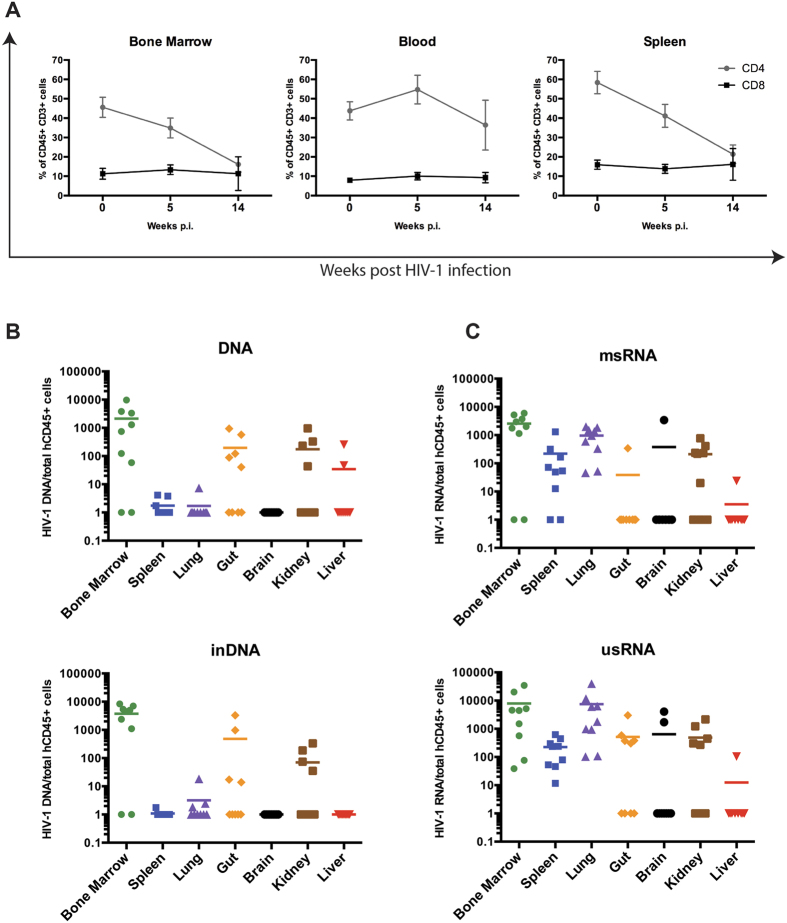
HIV-1 detection in humanized mice tissues. Humanized NSG mice were infected with HIV-1_ADA_; and after 5 week of infection, plasma viral load was assessed. First, (**A**) Percentages of CD4+ and CD8+ T cells were determined by flow cytometry from the total human CD45+ CD3+ gate. This analysis was performed at pre- (0) and post- infection (5 and 14 weeks) by flow cytometry. Then (**B**) total viral DNA and inDNA and (**C**) viral RNA (msRNA and usRNA) levels were determined by semi-nested real time PCR in bone marrow, spleen, lung, gut, brain, kidney and liver tissues. The figures represent nucleic acid viral copies, DNA or RNA, per 10^6^ cells normalized to human CD45+ cells, as described in methods.

Viral load determinations in plasma confirmed infection in humanized mice beginning four weeks after HIV-1 exposure. Animals containing plasma virus ranging from 10^3^ to 10^7^ viral copies/mL were selected for the study. Peripheral blood viral loads and percentage of CD4+ T cells, at the time of sacrifice, for all the mice included in this study are listed in Supplementary Table 2. After 14 weeks post-infection, we observed significant depletion of CD4+ T cells in bone marrow, blood and spleen. The CD4+T cell levels were reduced by 16.1% ± 3.9, 36.4% ± 12.8 and 21.3% ± 4.7 from 45.6% ± 5.1, 43.8% ± 4.7 and 58.4% ± 5.7, respectively. CD8+ T cells showed no significant change (Fig. 2A). Nine mice with comparable parameters of engraftment were selected for tissue viral analyses at five weeks following HIV-1_ADA_ infection. Bone marrow, spleen, lung, gut, brain, kidney and liver tissues were collected for viral quantification, as explained in methods. Total HIV-1 DNA, which does not distinguish between integrated and non-integrated DNA, and integrated DNA (in DNA) as well as HIV-1 multi-spliced RNA (msRNA) and unsliced RNA (usRNA) levels in cells or tissues of bone marrow, spleen, lung, kidney and gut were readily quantified by semi-nested qPCR with comparatively low copy numbers in spleen and kidney (Fig. 2B,C). In general, infected animals with high plasma viral load showed high levels of viral DNA and RNA. Viral RNA was also detected in brain and liver but only in a few animals and at low copy numbers. Species of msRNA that encode tat and rev proteins were linked to productive infection. Levels of viral usRNA and integrated DNA best reflect the pool of latently infected cells. While our assay detected viral infection in bone marrow cells, it is noteworthy that spleen contained lower levels of viral RNA compared to bone marrow cells perhaps providing evidence for viral infection in this tissue site. More extensive analysis will be done in future studies.

### Persistent HIV-1 infection affects the numbers and distribution of CD34+ progenitor, monocyte-macrophage and dendritic cells

**Figure 3:**
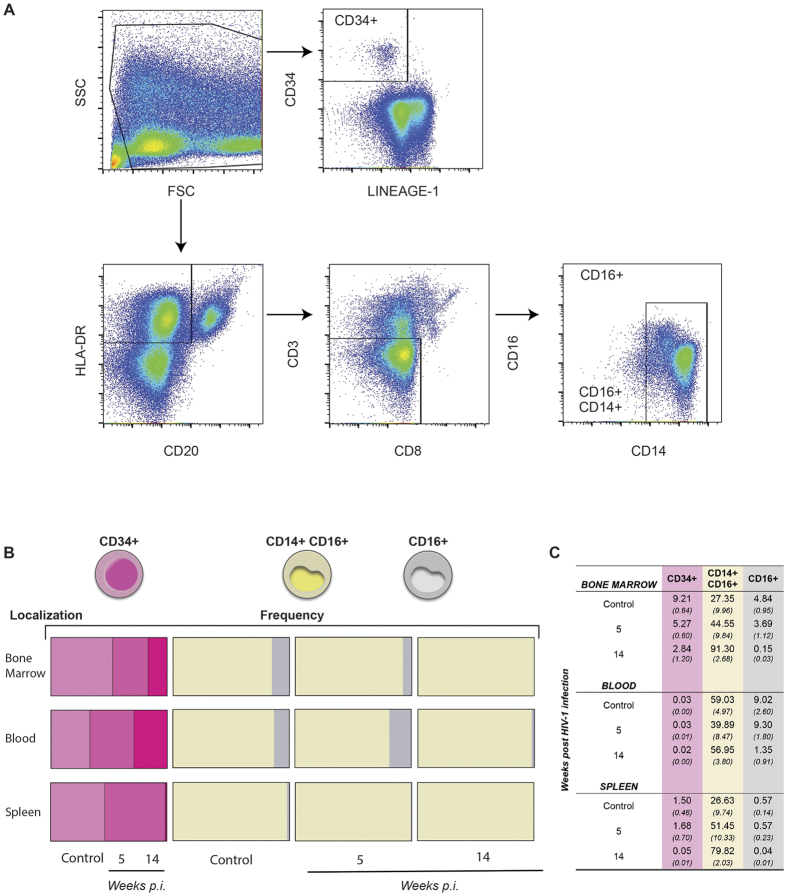
Identification of immune cell subsets. Multicolor flow cytometric analysis was performed on bone marrow, whole blood and spleen tissues. (**A**) Cells from each tissue were incubated with labelled antibodies for the identification of CD34, Lineage-1, CD19, HLA-DR, CD3, CD8, CD14 and CD16 cellular markers. Monocytes/Macrophages were identified as CD3−/CD20−/CD8−/HLA-DR+/CD14+/CD16+ and DC as CD3−/CD20−/CD8−/HLA-DR+/CD14−/CD16+. Lin- CD34+ progenitor cells were determined by the expression of CD34 marker, excluding all Lineage-1 cells, as shown in the histogram. (**B**) Representation of frequencies for human Lin- CD34+, monocytes/macrophages CD14+ CD16+ and dendritic cells CD14− CD16+ from bone marrow, blood and spleen origins. Cellular debris was excluded by their light-scattering characteristics, and dead cells were excluded by UV fluorescence using a live/dead fluorochrome (Life Technology). Data were analyzed with FlowJo software. (**C**) Frequencies of CD34+, monocytes/macrophages and dendritic cells during HIV-1 infection.

To determine how immune cell phenotypes change during the course of infection in humanized mice, we collected bone marrow, blood and spleen samples from uninfected and infected mice from 5 to 14 weeks post-infection (Supplementary Table 2). We developed flow cytometric gating strategies for clustering human Lineage negative (Lin-) CD34+ cells, mononuclear phagocytes (MP; CD14+ CD16+ monocyte-macrophages and CD14− CD16+ dendritic cells) and memory and regulatory CD4+ T cells (Fig. 3A) by detection of cellular markers as explained in methods. Cell phenotypes were altered during the course of HIV-1 infection compared with uninfected control animals (Fig. 3B,C). Lin- CD34+ cells decreased in numbers in bone marrow (2.84% ± 1.2) and spleen (0.05% ± 0.01) at 14 weeks post infection compared to uninfected controls (9.21% ± 0.84 and 1.5% ± 0.46), respectively. In contrast, monocyte-macrophages increased in frequency during viral infection, showing values in bone marrow and spleen of at least three times higher than the uninfected controls. This suggests that the virus infection induces a higher turnover of this cell population. In contrast, dendritic cells (CD14− CD16+) were reduced in bone marrow, blood and spleen at 14 weeks (Fig. 3C). All changes in cell phenotype were presented gradually from acute infection (5 weeks post infection) to chronic infection stage at 14 weeks post-infection as stocked bars showing frequency of each cell type (Fig. 3B) and in a table (Fig. 3C).

### Altered memory and regulatory CD4+ T cell phenotypes following HIV-1 infection

**Figure 4:**
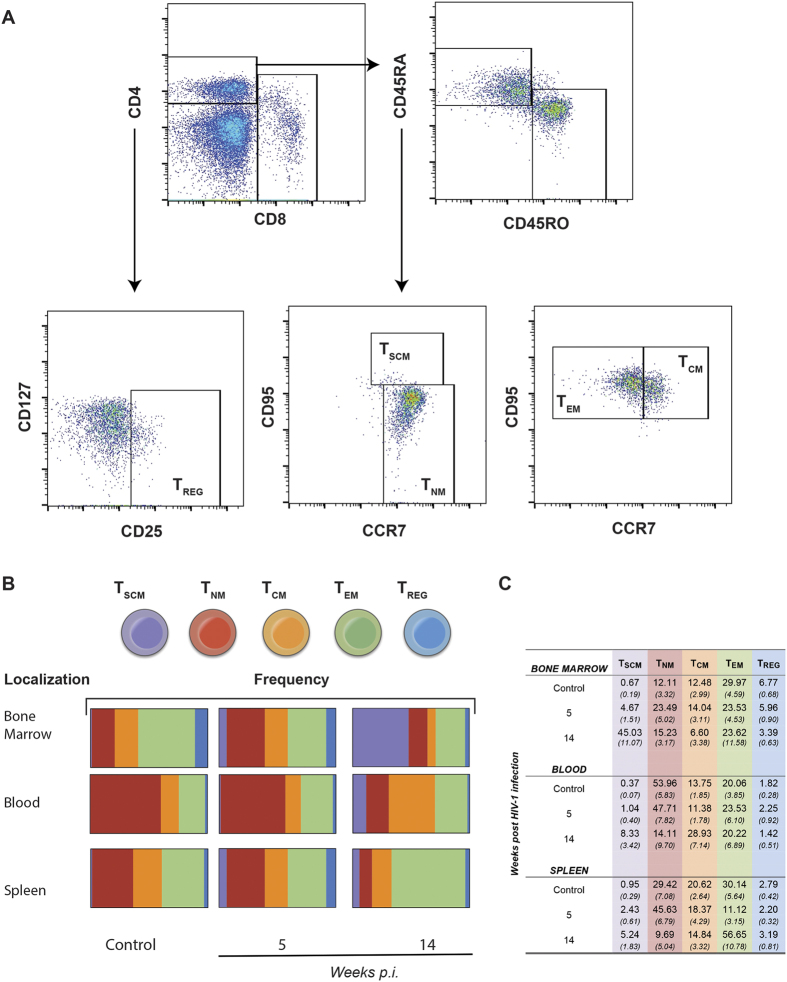
Frequencies of CD4+ memory and T_REG_ phenotypes during progressive HIV-1 infection in humanized mice. The T cells immune phenotypes were evaluated during acute (5 weeks) and chronic (14 weeks) infections in blood, spleen and bone marrow tissues. (**A**) Cell suspensions were labelled with anti-human monoclonal antibodies (mAb) targeting the following cell-surface markers: CD45, CD3, CD19, CD4, CD8, CD25, CD127, CD45RA, CD45RO, CD95, CCR7 (all from BD Biosciences). Histograms show the strategy for identification and isolation of CD4+ memory T cells (T_SCM_, T_NM_, T_CM_ and T_EM_) and T_REG_. All acquisitions were performed on a LSRII flow cytometer (Beckman Coulter). Cellular debris was excluded by their light-scattering characteristics, and dead cells were excluded by UV fluorescence using a live/dead fluorochrome (Life Technology). (**B**) Schematic description of the frequencies of T_SCM_, T_NM_, T_CM_ and T_EM_ and T_REG_ during HIV-1 acute and chronic infection in a humanized mice. Frequencies of immune cells were detected by flow cytometry following the strategies for gating and detecting each specific population, as described above. All data were analyzed by FlowJo software. (**C**) Frequency of memory and T_REG_ populations in HIV-1 infected humanized mice.

A broader analyses of human T cell subsets were performed (Fig. 4A) that included CD4+ stem cell memory, naïve memory, central memory, effector memory (T_SCM_, T_NM_, T_CM_, T_EM_) and regulatory T cells (T_REG_) respectively. These CD4+ T cell populations changed in numbers and distribution during the course of viral infection. This was seen in bone marrow, blood and spleen from 5 to 14 weeks (Fig. 4B). T_SCM_ were at the limit of detection in uninfected animals but increased in numbers as a consequence of virus infection. These cells were highest in frequency in bone marrow at 14 weeks (45.03% ± 11.07). Inversely, T_NM_ were reduced in frequency in both blood (to 14.11% from 53.96%) and spleen (to 9.69% from 29.42%) but remained unchanged in bone marrow. T_CM_ and T_EM_ populations showed more limited changes in frequencies. Interestingly, T_REG_ in bone marrow declined during viral infection by week 14. They were 3.39% ± 0.63 and 6.77% ± 0.68 in infected and uninfected controls, respectively (Fig. 4C).

### Identification of virus-infected cells in the bone marrow and spleen

**Figure 5:**
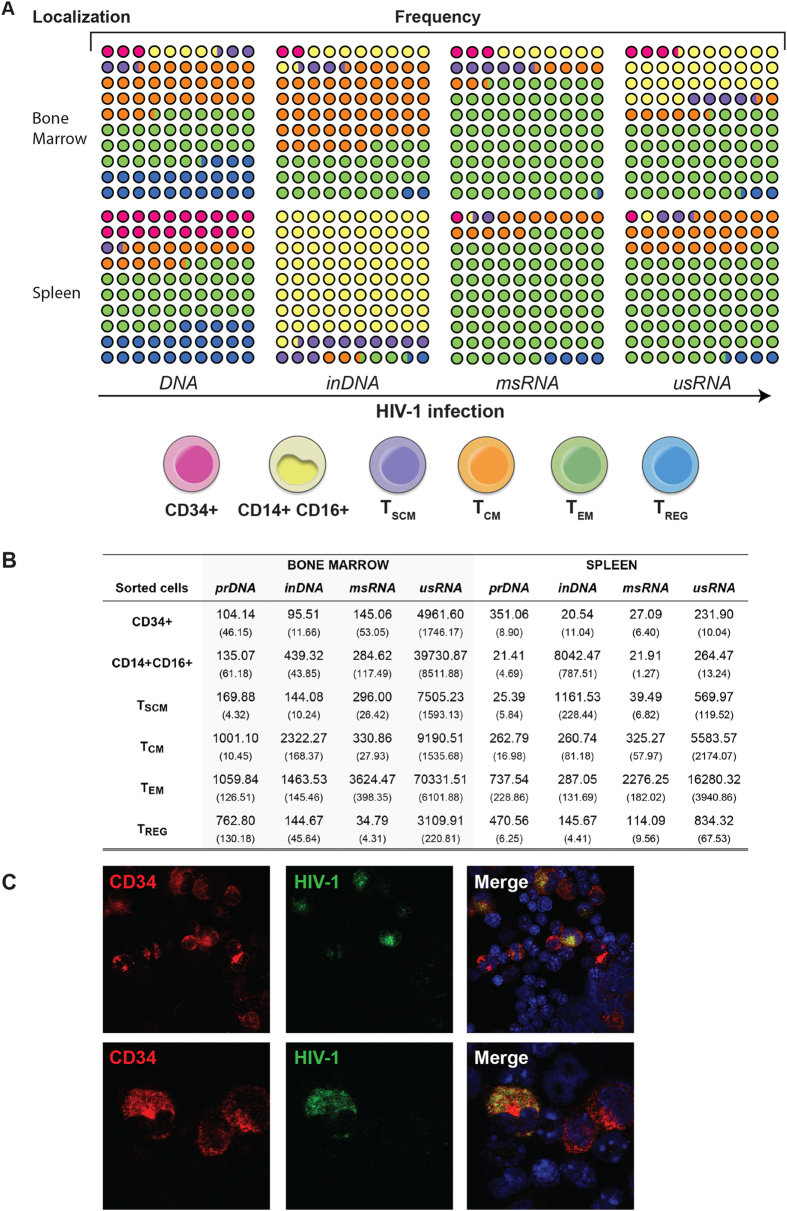
Defining the levels and species of HIV-1 infection in immune subsets from bone marrow and spleen. Tissues were collected and cells in suspension were incubated with anti-human CD45 magnetic beads for isolating human CD45+ cells previous to FACS. Specific antibodies were applied and cells were sorted following the same procedure as described for immunophenotyping. Sorted cells from eleven weeks HIV-1 infected humanized mice were used for detecting both total viral DNA and inDNA and RNA (msRNA and usRNA). (**A**) Sorted Lin- CD34+, Monocyte-macrophages CD14+ CD16+, CD4+ T_SCM_, CD4+ T_CM_, CD4+ T_EM_, and CD4+ T_REG_ cells were processed for DNA and RNA isolation and examined by semi-nested qPCR. Samples were quantified by the standard curve method using serial dilutions of viral standards (ACH2+ cells). Detection of total HIV-1 DNA, inDNA, msRNA and usRNA were achieved by using specific primers and probes as described in methods and in supplementary table 1. Dots and colors are representations for the frequency of viral DNA and RNA in different cell populations from bone marrow and spleen tissues. (**B**) Viral DNA and RNA quantification in bone marrow and spleen of HIV-1 infected humanized mice. The numbers indicate nucleic acid viral copies, DNA or RNA, per 10^4^ cells normalized to human GAPDH+ cells, as described in methods. Numbers between parentheses indicate the SEM. (C) Bone marrow cells from HIV-infected humanized mice were stained for the detection of human CD34+ cells (red) and HIV-p24 (green) and examined by confocal microscopy. Upper panel represent a picture at 400× original magnification and lower panel represent a different picture captured at 600X magnification. Representative images are shown from two HIV-infected humanized mice. Images were captured using a Zeiss confocal microscope.

We next sought to identify the viral cell subsets in HIV-1 humanized mice. Here, we isolated the human cells by fluorescence activated cell sorting (FACS) to Lin- CD34+ cells, monocyte-macrophages CD14+ CD16+, CD4+ T_SCM_, T_CM_, T_EM_ and T_REG_ from bone marrow and spleen. For these sorted cells, qPCR was performed for cell-associated HIV-1 total DNA, inDNA, msRNA and usRNA. msRNA species is linked to productive infection while usRNA and inDNA for latently infected cells. Infection of sorted cell populations, mentioned above, was observed at varied frequencies (Fig. 5A) as supported by independent measures of viral DNA and RNA species. There was a high frequency of viral RNA in monocyte-macrophages CD14+ CD16+, T_CM_ and T_EM_ from bone marrow and spleen. Total viral DNA and integrated viral DNA were found mainly in monocytes/macrophages, T_CM_, T_EM_ and T_REG_. The levels for viral DNA or RNA of specific cell populations are shown in Fig. 5B. Viral msRNA was detected at high levels in T_EM_ from bone marrow (3624.47 ± 398.35) and spleen (2276.25 copies ± 182.02). Interestingly, viral inDNA was observed principally in monocyte-macrophages from spleen (8042.37 ± 787.51), with low levels of HIV RNA in the same population. Viral inDNA was also abundant in T_CM_ and T_EM_ cells from bone marrow (2322.27 ± 168.37 and 1463.53 ± 145.67, respectively). Data from 11 weeks post infection are shown, as the numbers of sorted cells were limited at other time points.

### Infection of CD34+ hematopoietic bone marrow progenitor cells.

Lin- CD34+ cells were shown to be HIV-1 infected by assay of copies of viral RNA and DNA from bone marrow niche (Fig. 5B). To confirm these observations, we performed immunofluorescence analysis by confocal microscopy to visualize infected CD34+ cells from bone marrow of HIV-1 infected humanized mice. Figure 5C confirms infection of bone marrow cells from HIV-1 infected mice and supporting the virus’ ability to infect Lin- CD34+ hematopoietic progenitor cells. This supports the idea that a range of divergent cellular subsets are HIV-1 infected in this mouse model of human disease.

Overall, our findings describe the cellular targets for HIV-1 infection in a humanized mouse model system and gives important information about cellular subsets that need to be targeted when developing therapies in order to maximize viral clearance.

## Discussion

Establishing the cellular sites for HIV-1 infection during persistent viral infection is pivotal to elucidate how and where infection can continue despite a vigorous host immune response. Low levels of viral replication persist in a range of cell populations. While this has been demonstrated in human pathological tissues and in SIV infected rhesus macaques^17^,^31^,^32^, there has been no rigorous studies performed in cells in humanized mice^9^,^27^,^33^,^34^. The nature and composition of the HIV target cells and cell dynamics during infection have not yet been defined. Also, the characteristics of infection and specific viral life cycle changes are still unknown. Such studies have been hampered by the limited availability of tissue from individuals at varying disease stages or by whether SIV infections truly paralleled what is present in a human host^35^,^36^,^37^,^38^. In humanized mice, available techniques lacked both cell specificity and sensitivity for low copy viral DNA detection especially when small numbers of cells were made available^39^,^40^. To these ends, larger numbers of humanized mice were developed; and cells were pooled for analyses. Combined with flow cytometry, qPCR, FACS for cell separation and confocal microscopy, these assays permitted unique insights into cellular pools of infection and changes in cell turnovers during persistent viral infection. Methodology following previous report for the identification and quantification of viral RNA and DNA in specific sorted cell populations served as a tool for the understanding the characterization of infection dynamics in a variety of lymphocyte and monocyte-macrophage populations that can be considered as target cells. Interestingly, the bone marrow appeared as a major tissue site for infection where monocyte-macrophages and dendritic cells were principal cellular targets. The unique feature of this virus-cell interaction was that the viral life cycle was found to be completed with readily demonstrable viral proteins expressed. CD4+ T memory, stem and regulatory cells were the prominent infected cell type in the blood and peripheral lymphoid tissue, notably the spleen. Such results are consistent with human findings^8^,^41^,^42^. Notably, HIV-1 infection is associated with variable disease patterns. Some individuals develop severe immune suppression in less than one year; whereas, others develop disease in ten, reflecting in part the tempo of viral growth and the cell prominence for infection. Those infected individuals who maintain vigorous virological control with normal CD4+ T cells numbers for decades or more are elite controllers^43^,^44^,^45^,^46^. Here, genetic control of the virus life cycle in cell targets revolves around a range of transcriptional factors.

Notably, HIV-1 primary targets are memory CD4+ T cells with monocyte-macrophages and dendritic cells rarely infected^10^,^47^,^48^. All can gain entry in lymphoid tissues, including the gut, lymph nodes, spleen and the genitourinary system amongst other sites, where virus establishes long-term infection^37^,^49^. Moreover, HIV-1 nucleic acids are also seen in perivascular macrophages, parenchymal microglia and astrocytes. This can occur before the development of any pathological signs of encephalitis as seen by laser microdissection from brain tissue of persons who died in the presymptomatic disease stage. Such findings demonstrate that multiple viral sites for infection are operative, and the bone marrow is a source of infection from which progenitor cells and monocyte-macrophages would traffic virus before and during disease^50^,^51^,^52^.

HIV-1-specific CD4+ and CD8+ T cell responses are generally regarded as the backbone of antiviral immune activity as they define the adaptive immune response elicited against HIV-1^53^. However, in chronically infected patients, virus persists despite ART^17^,^54^,^55^. The role of CD4+ T memory cells (including memory stem cells) is well known during all stages of HIV-1 infection. These cells are the most prominent targets for virus that contribute to and promote long-term viral persistence^8^,^42^. Memory T cells constitute one of the most abundant lymphocyte population in the body. Their role mainly derives from mouse studies and how phenotype changes occur in humans may change based on the host microenvironment and susceptibility of infections^56^. In the current study, we sought to define immune cell populations that included CD34+, CD14+ CD16+, CD14− CD16+, CD4+ memory and regulatory cells actively infected with HIV-1 using a humanized mouse model of human disease. We posit that what was seen in this report holds parallel to those targets known to persist in humans during drug treatment. We also posit that monocyte-macrophages could contribute to the development of long-lived sites of infection and can be used as a therapeutic target in eradicating virus either through cell based drug delivery or by their abilities to closely interact with other CD4+ target cells. Interestingly, we found that bone marrow macrophages were a significant viral source. This was not seen in lymphoid tissues such as the spleen.

## Materials and Methods

### Animals

New born NSG mice were transplanted with human CD34+ stem cells, obtained from fetal liver, as previously described^15^,^57^. Animals were housed under pathogen-free conditions in accordance with ethical guidelines for the care of laboratory animals at the National Institutes of Health and the University of Nebraska Medical Center (UNMC). All experimental protocols were approved by UNMC’s Institutional Animal Care and Use Committee ensuring the ethical care and use of laboratory animals in experimental research. After twelve weeks of human CD34+ HSC transplantation, mice were selected based on human CD45+ cells counts in peripheral blood and divided in two groups for HIV-1 infection and for uninfected control. At different time points of infection (five, eleven and fourteen weeks), animals were sacrificed for further cell phenotyping and virus detection in tissues and in isolated cell populations.

### HIV-1 infection

Animals for HIV-1 infection group were infected with HIV-1_ADA_, by intraperitoneal injection, at 10^4^ TCID_50_, at twelve-fifteen weeks of age. HIV-1_ADA_ was prepared by propagating on monocyte-derived macrophages in DMEM medium and was handled under modified biosafety level-3 conditions, as previously described^58^.

### Quantification of plasma HIV-1 RNA

Peripheral blood samples were collected into ethylene diamine tetra acetic acid (EDTA)-coated tubes from the facial vein, and plasma was separated by centrifugation. The quantification of HIV-1 RNA in the plasma of the virus-infected humanized NSG (hNSG) mice collected at 4 weeks post-infection was performed using the Taqman Analyzer according to the manufacturer’s instructions (Roche Diagnostics, Indianapolis, USA).

### Peripheral blood and tissue collection

Animals from the uninfected control group and HIV-1 infected group were sacrificed to analyze the cell phenotypes in peripheral blood, bone marrow and spleen tissue samples. Peripheral blood samples were collected into EDTA-coated tubes by cardiocentesis at the time of euthanasia. Spleen and bone marrow were collected on 5% FBS/RPMI medium and then processed to obtain single cell suspensions for cell phenotyping, sorting and viral quantification. Single cells from spleen and bone marrow of hNSG HIV-1-positive mice were split into two aliquots: one for cell phenotyping and cell sorting, and the other one was pelleted and stored at −80 °C for viral quantification. Other tissues like lymph nodes, lung, gut, liver and brain from hNSG HIV-1 animals were also collected and stored at −80 °C for quantification of viral copies.

### Flow cytometry

For cell phenotyping, 100–200 μl of whole blood or small amounts (1–2 × 10^5^ cells) of spleen and bone marrow cell suspensions were incubated for 30 min with monoclonal antibodies for the identification of T cell subtypes, hematopoietic progenitor and monocytes/macrophage cells, following standards procedures for surface receptors staining by flow cytometry. Human monoclonal antibodies against CD45, CD3, CD4, CD8, CD45RA, CD45RO, CD95 and CCR7 were used for the identification of different memory T cells. Memory CD4+ T cells were subtyped as stem cell memory (T_SCM_), naïve memory (T_NM_), central memory (T_CM_) and effector memory (T_EM_) with the expression of CD45RA+/CCR7+/CD95+, CD45RA+/CCR7+, CD45RO+/CCR7+ and CD45RO+/CCR7−, respectively, from CD45+/CD3+/CD4+ gate. Moreover, CD4+ regulatory T cells (T_REG_) were classified as CD127+^low^/CD25+, hematopoietic progenitor cells CD34 as Lineage- CD34+, monocyte/macrophages as CD3−/CD20−/CD8−/HLA-DR+/CD14+/CD16+ and dendritic cells as CD3−/CD20−/CD8−/HLA-DR+/CD14−/CD16+. Flow cytometric acquisition and sample analysis were performed on an LSRII flow cytometer driven by the FACSDiva software package (BD Biosciences, California, USA). All antibodies were obtained from the same source.

For cell sorting, spleen and bone marrow single cell suspensions from hNSG HIV-1 animals were used. One aliquot of each tissue was pooled and isolated for human CD45+ cells using magnetic beads, then cells were incubated with a different panel of antibodies as described above and sorted by FACS to isolate specific memory T cells populations as described above. After 30 min of incubation, CD45RA+/CCR7+/CD95+ T_SCM_, CD45RO+/CCR7+ T_CM_, CD45RO+/CCR7− T_EM_ and CD25+ T_REG_ CD4+ T cells were live-sorted in a specifically designated biosafety cabinet using a FACS Aria cell sorter (BD, New Jersey, USA). During sorting, cells were collected in 5% FBS/RPMI medium, on ice, then pelleted by centrifugation and stored at −80 °C for further viral quantification analysis. Cell sorting was performed by the Flow cytometry Core Facility at UNMC. Analysis of all the acquired data was performed using FlowJo Version 10.08 software (TreeStar).

### Nucleic acid extraction

Total nucleic acids (RNA or DNA) from tissue or from cells were acquired from the spleen, bone marrow, lymph node, lung, gut, liver, brain or sorted CD4+ memory, and T_REG_ were extracted using a Qiagen Kit (Qiagen, Hilden, Germany) according manufacturer’s instructions. Aliquots of eluted samples were used for qPCR tests or frozen at −80 °C until further processing. Nucleic acids from HIV-1 infected cell line ACH2 served as standard positive control. Cell-associated HIV-1 RNA and DNA were quantified by semi-nested real time PCR.

### qPCR for total HIV-1 DNA

For semi-nested real-time PCR for total HIV-1 DNA, the eluted cellular DNA or DNA standards were directly subjected to two rounds of PCR amplification. We followed the protocol as described by Pasternack *et al.*^59^ with some modifications for the amplification of a region within the HIV-1 *gag* gene. The first round of the PCR was performed on a conventional PCR machine (T100 Thermal Cycler, Biorad, California, USA) in 25 μl of PCR master mix containing 5 μl of template and 50 ng each of both primers. The PCR settings were as follows: 94 °C for 3 min, followed by 15 cycles of 94 °C for 30 s, 55 °C for 30 s, and 72 °C for 1 min. The product of the first PCR was subsequently used as a template in the second semi-nested real-time, PCR amplification performed on the ABI Prism 7000 real-time PCR machine (Applied Biosystems, Massachusetts, USA) using TaqMan detection chemistry. A total of 2 μl of the first PCR product was diluted to 50 μl with PCR master mix containing 0.2 uM concentrations of each of both primers and 0.2 uM TaqMan dual-labeled fluorescent probe. Real-time PCR settings were as follows: 50 °C for 2 min, then 95 °C for 10 min, followed by 50 cycles of 95 °C for 15 s and 60 °C for 1 min. The amplicon sizes were 221 bp for the first PCR and 83 bp for the second (real-time) PCR. ACH2 cells (8 × 10^5^) containing one integrated copy of HIV-1 per cell were used in triplicate as standards with cell and HIV copy numbers ranging in serial 10-fold dilutions from 10^5^ to 10^2^ DNA copies/ reaction.

The detection of total viral DNA in our assay does not discriminate between integrated and unintegrated forms of HIV-1. It provides a relative quantification to a standard curve.

### qPCR for alu-gag integrated DNA

inDNA provirus was quantified using an adapted *alu*-PCR assay as described by Agosto *et al.*^60^, with modifications for the second round of PCR, following similar methods as Pasternack *et al.*^59^. Briefly, samples underwent a first-round PCR amplification (95 °C for 2 min; 20 cycles of 95 °C for 15 s, 50 °C for 15 s, and 72 °C for 150 s) using 100 nM *alu* and 600 nM *gag* reverse primers. Five to ten μl of the first-round product was amplified in a nested protocol using the assay for HIV-1 *gag* gene (second PCR primers and probe), as described above. A first-round PCR with 3 replicates using only the *gag* reverse primer (*gag* only) acted as a background unintegrated control. Serially diluted integration site standards were used to construct a standard curve for each plate. Integration levels per cell were calculated by subtracting *gag*-only signals from the *alu-gag* quantification.

### qPCR for viral RNA

Semi-nested real-time PCR on HIV-1 RNA was performed as described^59^. The eluted cellular RNA was first subjected to DNase treatment to remove HIV-1 DNA, which could interfere with the quantitation. For RT assay, we used random hexamers as primers and SuperScript III (Invitrogen, Massachusetts, USA) at 42 °C for 60 min according to the manufacturer’s instructions. cDNA was divided into two portions: one was used in the usRNA assay, and the other was used in the msRNA assay. Two rounds of PCR were performed under the same PCR conditions as described above for the total viral DNA assay. For the usRNA assay, real-time PCR was run for 45 cycles; and for the msRNA assay, real-time PCR was run for 50 cycles. For the usRNA assay, the same primers and fluorescent probe were used as for the total viral DNA assay. The first PCR of the msRNA assay was performed with primer pairs that amplify msRNA species encoding the Tat and Rev proteins, as previously described^59^. Semi-nested real-time PCR of the msRNA assay was performed with the primers and the TaqMan fluorescent probe. The amplicon sizes were 171 bp for the first PCR and 115 bp for the second (real-time) PCR of the msRNA assay.

For sorted cells, levels of HIV-1 DNA and RNA were normalized to the expression of the housekeeping gene human GAPDH (Life Technology, California, USA). For viral detection in tissues of infected humanized mice, expression levels were normalized to human CD45 gene (Life Technology, California, USA). All primers sequences used in this study are listed in Supplementary Table 1.

### Immunofluorescence and confocal imaging

For immunofluorescence staining, bone marrow cells were collected from the bones of infected humanized mice and cytospin slides were prepared immediately after cell collection. Cells were fixed with 3.7% formaldehyde at room temperature for 20 min followed by PBS wash. Fixed cells were permeabilized with 0.5% Triton X-100 in PBS and then blocked with 5% bovine serum albumin (BSA) in PBS for 30 min. Cells were washed and sequentially incubated with primary antibody against HIV-1 p24 (Dako, California, USA) and anti-human CD34 (Abcam, Massachusetts, USA) for 1 hour then washed 3 times with PBS. Secondary antibodies conjugated with Alexa Fluor 488 or Alexa Fluor 594 dyes (Life Technologies-Molecular Probes, New York, USA) were applied against the primary antibody isotype and incubated at room temperature for 1 hour then washed 3 times with PBS. Slides were covered in ProLong Gold AntiFade reagent with 4′,6-diamidino-2-phenylindole (DAPI) (Life Technologies-Molecular Probes, New York, USA) and imaged using a 40× oil lens on a LSM 710 confocal microscope (Carl Zeiss Microimaging, Inc.; New York, USA).

## Additional Information

**How to cite this article**: Araínga, M. *et al.* HIV-1 cellular and tissue replication patterns in infected humanized mice. *Sci. Rep.* **6**, 23513; doi: 10.1038/srep23513 (2016).

## Supplementary Material

Supplementary Information
