# Supplementary material for: HIV-1 cellular and tissue replication patterns in infected humanized mice
Source: Sci Rep. 2016 Mar 21;6:23513. doi: 10.1038/srep23513 (PMC4800734; doi:10.1038/srep23513)
Supplement: Supplementary Information [file srep23513-s1.pdf]

HIV-1 cellular and tissue replication patterns in infected humanized mice

Mariluz Araínga, Hang Su, Larisa Poluektova, Santhi Gorantla and Howard E. Gendelman

Supplementary Information

Supplementary Table 1. Oligonucleotide primers and probes

Assay	Genomic region	Type (a)	Sequence (5' – 3')
Viral DNA	*gag*	Sense [1-2]	TCAGCCCAGAAGTAATACCCATGT
usRNA		Antisense [1]	TGCTATGTCAGTTCCCCTTGGTTCTCT
		Antisense [2]	CACTGTGTTTAGCATGGTGTTT
		Probe	FAM-ATTATCAGAAGGAGCCACCCCACAAGA-TAMRA
Integrated	*alu*	Sense	GCCTCCCAAAGTGCTGGGATTACA
DNA	*gag*	Antisense	GTTCCTGC TATGTCACTTCC
msRNA	*tat/rev*	Sense [1]	CTTAGGCATCTCCTATGGCAGGAA
		Antisense [1-2]	GGATCTGTCTCTGTCTCTCTCTCCACC
		Sense [2]	ACAGTCAGACTCATCAAGTTTCTCTATCAAAGCA
		Probe	FAM-TTCCTTCGGGCCTGTCGGGTCCC-TAMRA

(a) 1, used for first PCR; 2, for second (real time) PCR

Supplementary Table 2. Plasma Viral Load (PVL) of HIV-1 infected humanized mice

Weeks p.i.	Animal ID	PVL (mL)	copies/mL	CD4 T cell (%)
5	219	230000	2.30E+05	20.90
5	221	9150	9.15E+03	17.10
5	222	1000	1.00E+03	36.70
5	2042	286696	2.87E+05	62.00
5	2045	1411	1.41E+03	67.40
5	2047	1271455	1.27E+06	69.00
5	2048	7635	7.64E+03	83.00
5	2049	1043000	1.04E+06	80.10
5	2050	38587	3.86E+04	65.80
5	842+	18900	1.89E+04	73.90
5	844+	2050000	2.05E+06	26.30
11	2226	13650	1.37E+04	37.20
11	2228	16475	1.65E+04	73.30
11	2205	2230000	2.23E+06	24.30
11	2214	1722500	1.72E+06	21.40
11	2215	1715000	1.72E+06	17.40
11	2216	70250	7.03E+04	53.10
11	2221	967500	9.68E+05	3.16
11	2234	1392500	1.39E+06	37.70
11	2236	562500	5.63E+05	22.60
11	2237	226500	2.27E+05	8.41
11	2244	5050000	5.05E+06	52.10
11	2245	1105000	1.11E+06	13.90
14	1924	377000	3.77E+05	18.10
14	1931	770000	7.70E+05	29.50
14	1932	955000	9.55E+05	23.60
14	1933	367000	3.67E+05	74.40
